# Transplantation of human dental pulp stem cells ameliorates diabetic polyneuropathy in streptozotocin-induced diabetic nude mice: the role of angiogenic and neurotrophic factors

**DOI:** 10.1186/s13287-020-01758-9

**Published:** 2020-06-16

**Authors:** Masaki Hata, Maiko Omi, Yasuko Kobayashi, Nobuhisa Nakamura, Megumi Miyabe, Mizuho Ito, Eriko Makino, Saki Kanada, Tomokazu Saiki, Tasuku Ohno, Yuka Imanishi, Tatsuhito Himeno, Hideki Kamiya, Jiro Nakamura, Shogo Ozawa, Ken Miyazawa, Kenichi Kurita, Shigemi Goto, Jun Takebe, Tatsuaki Matsubara, Keiko Naruse

**Affiliations:** 1 Department of Removable Prosthodontics, School of Dentistry, Aichi Gakuin University, Nagoya, Japan; 2 Department of Internal Medicine, School of Dentistry, Aichi Gakuin University, 2-11 Suemori-dori, Chikusa-ku, Nagoya, 464-8651 Japan; 3 Department of Orthodontics, School of Dentistry, Aichi Gakuin University, Nagoya, Japan; 4 Department of Pharmacy, Dental Hospital, Aichi Gakuin University, Nagoya, Japan; 5 Department of Periodontology, School of Dentistry, Aichi Gakuin University, Nagoya, Japan; 6 Division of Diabetes, Department of Internal Medicine, Aichi Medical University School of Medicine, Nagakute, Japan; 7 Department of Oral and Maxillofacial Surgery, School of Dentistry, Aichi Gakuin University, Nagoya, Japan

**Keywords:** Human dental pulp stem cells (hDPSCs), Diabetic polyneuropathy, Cell therapy, Regenerative medicine, Diabetes, NGF, VEGF

## Abstract

**Background:**

Dental pulp stem cells (DPSCs) have high proliferation and multi-differentiation capabilities that maintain their functionality after cryopreservation. In our previous study, we demonstrated that cryopreserved rat DPSCs improved diabetic polyneuropathy and that the efficacy of cryopreserved rat DPSCs was equivalent to that of freshly isolated rat DPSCs. The present study was conducted to evaluate whether transplantation of cryopreserved human DPSCs (hDPSCs) is also effective for the treatment of diabetic polyneuropathy.

**Methods:**

hDPSCs were isolated from human impacted third molars being extracted for orthodontic reasons. Eight weeks after the induction of diabetes in nude mice, hDPSCs (1 × 10^5^/limb) were unilaterally transplanted into the hindlimb skeletal muscle, and vehicle (saline) was injected into the opposite side as a control. The effects of hDPSCs were analyzed at 4 weeks after transplantation.

**Results:**

hDPSC transplantation significantly ameliorated reduced sensory perception thresholds, delayed nerve conduction velocity, and decreased the blood flow to the sciatic nerve in diabetic mice 4 weeks post-transplantation. Cultured hDPSCs secreted the vascular endothelial growth factor (VEGF) and nerve growth factor (NGF) proteins. A subset of the transplanted hDPSCs was localized around the muscle bundles and expressed the human VEGF and NGF genes at the transplanted site. The capillary/muscle bundle ratio was significantly increased on the hDPSC-transplanted side of the gastrocnemius muscles in diabetic mice. Neutralizing antibodies against VEGF and NGF negated the effects of hDPSC transplantation on the nerve conduction velocity in diabetic mice, suggesting that VEGF and NGF may play roles in the effects of hDPSC transplantation on diabetic polyneuropathy.

**Conclusions:**

These results suggest that stem cell transplantation with hDPSCs may be efficacious in treating diabetic polyneuropathy via the angiogenic and neurotrophic mechanisms of hDPSC-secreted factors.

## Background

Dental pulp stem cells (DPSCs) in the dental pulp cavity have high self-renewal and proliferation activity and multipotency in terms of differentiation, similar to bone marrow-derived MSCs [1]. DPSCs are expected to be a resource for regenerative medicine because they can be easily isolated during wisdom tooth extraction or premolar extraction for orthodontic reasons and because they maintain their differentiation and proliferation capacities even after cryopreservation [2, 3]. DPSCs secrete abundant factors, including angiogenic and neurotrophic factors, and have immunomodulatory effects in immune cells [3, 4]; these characteristics further suggest that DPSCs are suitable for cell transplantation therapy.

Diabetic polyneuropathy is the most common complication in diabetes and is observed in approximately 30–50% of diabetic patients [5]. The clinical symptoms of diabetic polyneuropathy, such as decreased sensation, numbness, prickling, stabbing, and pain, are varied and impair quality of life [6, 7]. The pathogenesis of diabetic polyneuropathy is mainly neuronal and vascular disorders [8]. Cultured schwann cells from both type 1 and type 2 diabetic mice showed lower production levels of neurotrophins, such as NGF and NT-3, than those from normal mice [9], and diabetic rats showed decreased NGF levels in the sciatic nerves [10, 11]. Vascular abnormalities have been observed in diabetic polyneuropathy, which suggests the existence of ischemic damage [12, 13]. However, the current therapies for diabetic polyneuropathy mainly aim to manage the symptom of pain in addition to improving glycemic control [6]. Curative therapies are needed based on the pathogenesis of diabetic polyneuropathy.

Cell therapy using stem or progenitor cells is considered an attractive treatment option because of the regenerative ability and abundant cytokine secretion of these cell types. We and others have found that the transplantation of endothelial progenitor cells (EPCs) and bone marrow-derived MSCs improved diabetic polyneuropathy, accompanied by augmentation of nerve conduction velocity and nerve blood flow [14–16]. We also demonstrated that the transplantation of cryopreserved DPSCs derived from rats and freshly isolated DPSCs equally improved diabetic polyneuropathy [3].

In the present study, we evaluated the efficacy of human DPSCs (hDPSCs) in treating diabetic polyneuropathy as well as the mechanism by which these cells ameliorate diabetic polyneuropathy. We examined whether transplantation of DPSCs derived from human third molars improved nerve conduction velocity, nerve blood flow, and sensation disorder in diabetic mice. To investigate whether transplanted hDPSCs are localized at the transplanted site and express secretomes, the expression of human genes was evaluated in the hDPSC-transplanted skeletal muscles of the mice. We further evaluated the involvement of angiogenic and neurotrophic factors in the therapeutic effects of hDPSC transplantation using neutralizing antibodies.

## Methods

### Isolation of hDPSCs

**Fig. 1:**
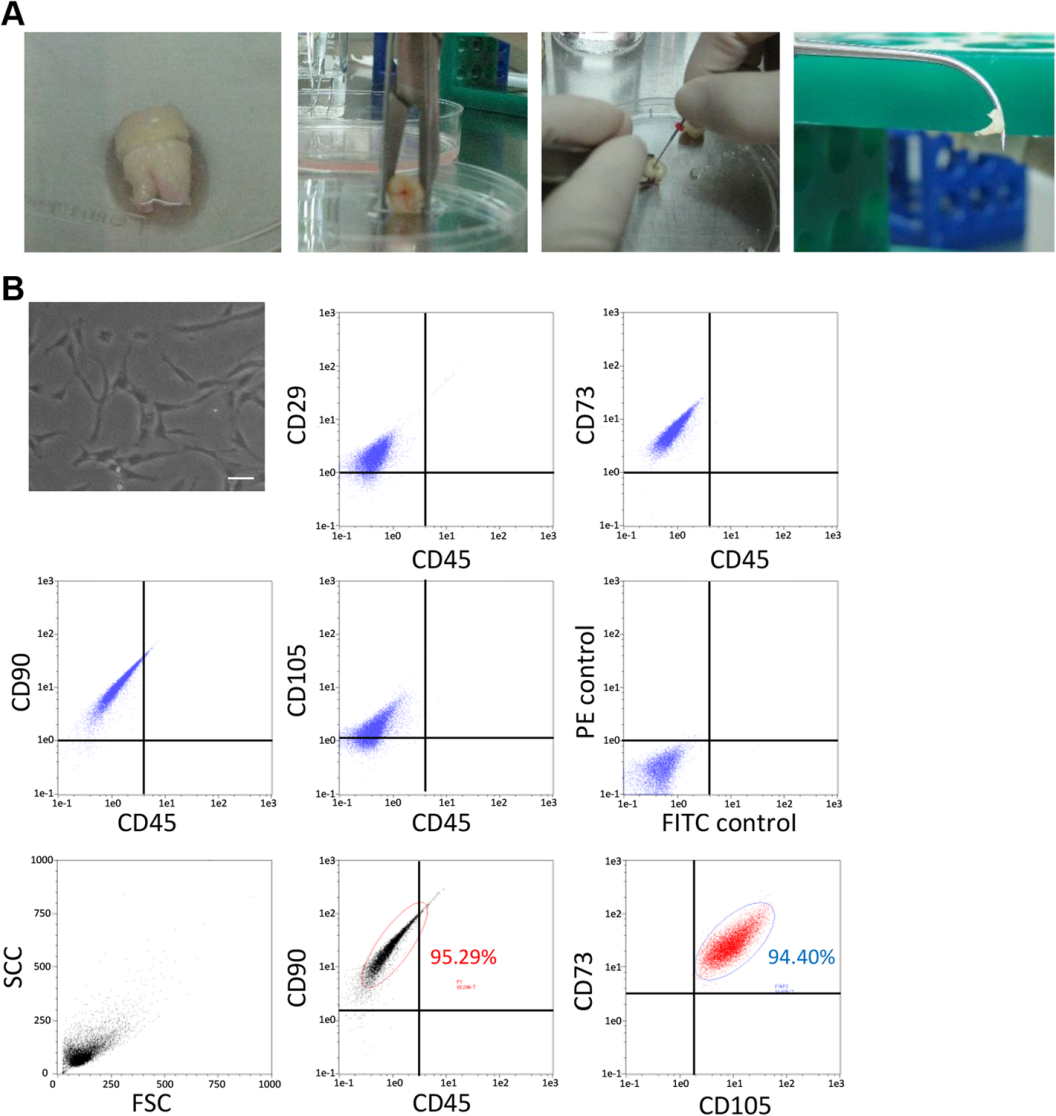
Characterization and differentiation of hDPSCs from human dental pulp tissue. **a** The dental pulp was extracted from the impacted third molars with forceps. **b** Cultured hDPSCs observed with a phase-contrast microscope. Bar = 100 μm. Representative flow cytometric histograms. Cell surface double staining was conducted with CD29, CD73, CD90, and CD105 antibodies and the CD45 antibody. CD90^+^ and CD45^−^ cells were selected, and CD73- and CD105-positive cell counts were determined

We collected human impacted third molars from 4 adults (13–23 years of age) undergoing tooth extraction for orthodontic reasons at Aichi Gakuin University Hospital. The dental pulp was extracted, and hDPSCs were isolated in phosphate-buffered saline (PBS) containing 0.1% collagenase and 0.25% trypsin (Fig. 1a). The hDPSCs were then cultured in Eagle’s medium with alpha modification (GIBCO Laboratories Inc., Grand Island, NY, USA) supplemented with 20% fetal bovine serum (GIBCO) [17]. At passage 3, the cells were cryopreserved with CELL BANKER (Amsbio, Cambridge, MA, USA) and stored in a freezer. For the experiments, cryopreserved hDPSCs were immediately thawed in a 37 °C water bath and washed with PBS three times. Recultured and expanded cryopreserved hDPSCs were used for the following experiments.

### Flow cytometric analysis

hDPSCs were analyzed via fluorescence-activated cell sorting (MACSQunat analyzer; Miltenyi Biotec, Bergisch Gladbach, Germany). The cells were specifically incubated with PE-conjugated mouse monoclonal antibodies against mouse CD29, CD73, CD90, and CD105 (Becton Dickinson, Franklin Lakes, NJ, USA) and FITC-conjugated mouse monoclonal antibodies against mouse CD45. Four-color analyses were performed with PE-conjugated mouse monoclonal antibodies against mouse CD90, APC-conjugated mouse monoclonal antibodies against mouse CD73, PerCP-Cy™5.5-conjugated mouse monoclonal antibodies against mouse CD105, and FITC-conjugated mouse monoclonal antibodies against mouse CD45. Isotype-identical antibodies served as controls. The data were analyzed with the MACSQuantify software (Miltenyi Biotec) [18].

### Animals and induction of diabetes

Six-week-old male nude mice (BALB/cAJcl-nu/nu) were obtained from Chubu Kagakushizai (Nagoya, Japan). Diabetes was induced in half of the mice by an intraperitoneal injection of streptozotocin (STZ; 150 mg/kg; Sigma Chemical, St Louis, MO, USA) [19]. Mice with blood glucose levels greater than 14 mmol/L were identified as diabetic and were used in the experiments. Mice not administered with STZ were bred as normal mice.

### Transplantation of hDPSCs

**Fig. 2:**
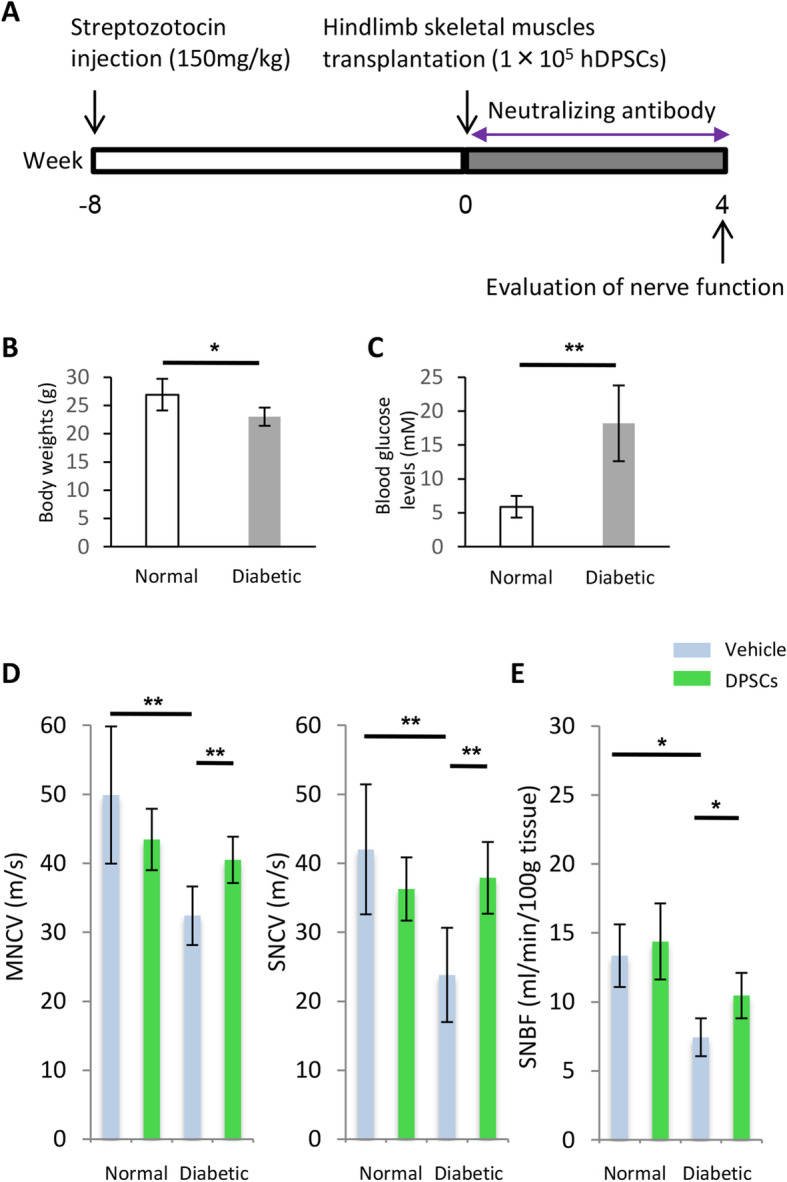
Transplantation protocol, nerve conduction velocity, and sciatic nerve blood flow. **a** Eight weeks after the injection of streptozotocin, 1 × 10^5^ hDPSCs were transplanted at 10 separate sites in the unilateral hindlimb muscle. **b** Body weights of nude mice. **c** Blood glucose concentration of nude mice. **d** The MNCV and SNCV of diabetic mice were significantly delayed compared with those of normal mice. Transplantation of hDPSCs ameliorated the delayed MNCV and SNCV. **e** Measurement of blood flow to the sciatic nerve was performed at 4 weeks after transplantation. The results are expressed as the mean ± SD (*n* = 4). *P < 0.05, **P < 0.01. MNCV, motor nerve conduction velocity; SNCV, sensory nerve conduction velocity; SNBF, sciatic nerve blood flow

Eight weeks after the induction of diabetes, hDPSCs (1 × 10^5^/limb) in 0.2 mL of saline were injected at 10 separate sites in the unilateral right hindlimb skeletal muscle (Fig. 2a). Vehicle (saline) was injected into the hindlimb skeletal muscle on the opposite side as a control. Four weeks after transplantation, the following measurements were performed.

### Nerve conduction velocity

Mice were anesthetized with isoflurane and placed on a heated pad in a room maintained at 25 °C, and the near-nerve temperature was maintained at 37 °C using a BAT-12 multipurpose thermometer (Bioresearch Co., Nagoya, Japan). The sciatic nerve motor nerve conduction velocity (MNCV) and the sciatic nerve sensory nerve conduction velocity (SNCV) were measured as previously described [3].

### Nerve blood flow

The blood flow to the sciatic nerve was measured by laser Doppler flowmetry (FLO-N1; Omegawave Inc., Tokyo, Japan). Mice were anesthetized with isoflurane, and the near-nerve temperature was maintained at 37 °C using a warming pad and a multipurpose thermometer. To measure sciatic nerve blood flow (SNBF), the thigh skin of each anesthetized mouse was cut along the femur, and an incision through the fascia was then carefully made to expose the sciatic nerve.

### Current perception threshold

To evaluate the nociceptive threshold, the current perception threshold (CPT) was measured using a CPT/LAB neurometer (Neurotron, Denver, CO). The electrodes for stimulation were attached to plantar surfaces. Each mouse was kept in a Ballman cage (Natsume Seisakusho, Tokyo, Japan) suitable for light restraint in the awake state. Transcutaneous nerve stimuli, consisting of three sine-wave pulses (at 5, 250, and 2000 Hz), were then applied to the plantar surfaces of the mice. The intensity of each stimulation was gradually increased automatically. The minimum intensity at which the mouse withdrew its paw was defined as the CPT. The CPT is reported as the mean of the values obtained from the fourth and fifth measurements.

### Tracking of transplanted hDPSCs in the hindlimb skeletal muscles

Four weeks after transplantation, the mice were killed by isoflurane. The gastrocnemius muscles were then removed from normal and diabetic mice and fixed with 4% paraformaldehyde overnight. To characterize the transplanted hDPSCs, frozen gastrocnemius muscle sections were observed by fluorescence microscopy. For this purpose, the muscles were embedded in a specific immersing solution (SCMM) in liquid nitrogen and cooled isopentane, and the frozen samples were cut serially into 5-μm-thick sections. The sections were subsequently mounted using adhesive film (Cryofilm type 1; Leica Microsystems, Wetzlar, Germany) and mounting medium (SCMM-R2; Leica Microsystems). The sections were then incubated with primary antibodies, including an anti-human nuclei antibody (Millipore). Human nuclei were detected using the Zenon Alexa Fluor 488 Mouse IgG1 Labeling Kit (Life Technology Co, Tokyo, Japan), and the sections were analyzed by fluorescence microscopy (Leica AF6000LX, Leica Microsystems).

### Human gene expression in the hindlimb skeletal muscles

Total RNA was extracted from frozen samples of gastrocnemius muscles using TRIzol Reagent (Invitrogen, Carlsbad, CA). Complementary DNA was synthesized from 1 μg of the RNA using ReverTra Ace (Toyobo, Osaka, Japan). Primers for the human vascular endothelial growth factor (VEGF), nerve growth factor (NGF), and β-actin genes were utilized for TaqMan Gene Expression Assays (Applied Biosystems, Foster City, CA). Samples were prepared with TaqMan Universal PCR Master Mix (Applied Biosystems), and polymerase chain reaction (PCR) products were observed by agarose gel (Wako, Osaka, Japan) electrophoresis with ethidium bromide (Sigma) staining.

### Protein levels of human VEGF and human NGF in serum and hDPSC culture supernatants

For hDPSC culture supernatants, hDPSCs were washed with PBS and maintained in Dulbecco’s modified Eagle’s medium (DMEM). After 24 h of incubation, the culture media were collected, and the protein concentrations of human VEGF and human NGF in serum and hDPSC culture supernatants were measured by ELISA kits (R&D Systems and Bioscience, Thebarton, South Australia) according to the manufacturer’s instructions. Samples were measured by a microplate reader (Spark; Tecan Trading AG, Männedorf, Switzerland).

### Capillary density/muscle bundle in the gastrocnemius muscles

The gastrocnemius muscles in paraffin were cut into 5-μm-thick sections for immunohistochemical staining. The sections were incubated overnight at 4 °C with the anti-PECAM-1 polyclonal antibody (Santa Cruz Biotechnology Inc., Dallas, TX, USA) diluted 1:500 and then stained using the Simple Stain Mouse System (Nichirei, Tokyo, Japan). Five fields from each section were randomly selected for the blind counting of capillary endothelial cells under a light microscope (× 200) by one skilled investigator to determine the capillary density.

### Inhibition of VEGF and NGF signals using neutralizing antibodies in vivo

To clarify the effects of VEGF and NGF secretion on hDPSC transplantation, diabetic mice were treated with a VEGF-neutralizing antibody (R&D Systems) and/or an NGF-neutralizing antibody (R&D Systems) using an Alzet osmotic pump (Durect Corporation, Cupertino, CA) (0.5 μg/mice/day) soon after hDPSC transplantation. Four weeks later, MNCV and SNCV were bilaterally measured.

### Statistical analysis

All group values are expressed as the mean ± standard deviation of the mean. Statistical analysis was performed by Student’s *t* test for comparisons of body weight and blood glucose between the two groups and by one-way ANOVA with Bonferroni correction for multiple comparisons. Differences were considered significant at *P* < 0.05.

## Results

### Characteristics of hDPSCs from human dental pulp tissue

hDPSCs cultured on a plastic dish exhibited typical spindle-shaped morphology, as determined by phase-contrast microscopy. Flow cytometric analyses with two-color immunofluorescence staining revealed that the hDPSCs were positive for CD29, CD73, CD90, and CD105 and negative for CD45. For multicolor analysis, the percentage of CD90^+^CD45^−^ cells was 95.29% and that of CD73^+^CD105^+^ cells gated on CD90^+^CD45^−^ cells was 94.40% (Fig. 1b).

### Body weights and blood glucose levels

At the end of the experiments (12 weeks after STZ injection and 4 weeks after hDPSC transplantation), compared with normal mice, the diabetic mice showed significantly decreased body weights (normal mice, 26.9 ± 2.8 g; diabetic mice, 23.0 ± 1.6 g; *P* < 0.05) and significantly increased blood glucose levels (normal mice, 5.9 ± 1.6 mM; diabetic mice, 18.2 ± 5.6 mM; *P* < 0.01) (Fig. 2b, c).

### MNCV, SNCV, and SNBF improvements induced by hDPSC transplantation

We evaluated the MNCV and SNCV at 4 weeks after hDPSC transplantation (Fig. 2d), revealing significantly reduced values on the vehicle-injected side of the diabetic mice compared with the normal mice. The impaired MNCV and SNCV were significantly restored on the hDPSC-transplanted side of the diabetic mice (*P* < 0.01).

SNBF was also reduced on the vehicle-injected side of the diabetic mice compared with the normal mice (Fig. 2e). Transplantation of hDPSCs significantly augmented the SNBF on the hDPSC-injected side of the diabetic mice at 4 weeks after transplantation (*P* < 0.05). hDPSC transplantation did not affect the MNCV, SNCV, or SNBF in normal mice.

### Effects of hDPSC transplantation on reduced sensory perception in the diabetic mice

**Fig. 3:**
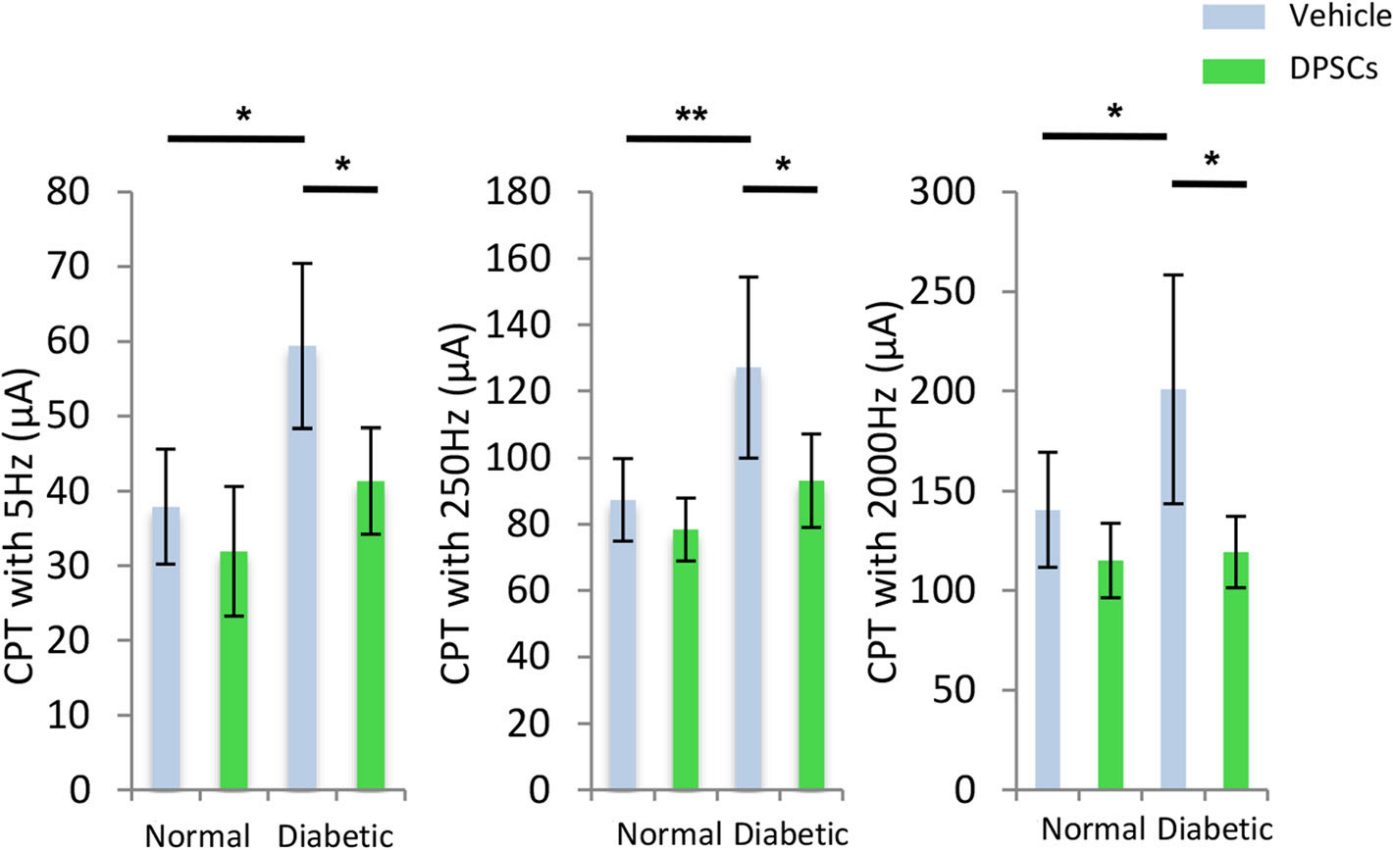
Sensory nerve function. The current perception thresholds (CPTs) at 5 Hz, 250 Hz, and 2000 Hz stimulated the C fiber, Aδ fiber, and Aβ fiber, respectively. The CPTs were measured with a neurometer 4 weeks after hDPSC transplantation. The CPTs for all frequencies were significantly increased in the diabetic group, and the impairments were ameliorated on the transplanted side. The results are expressed as the mean ± SD (*n* = 4). *P < 0.05, **P < 0.01

We assessed the sensory functions based on the CPT (Fig. 3). CPTs at 5, 250, and 2000 Hz expressed the sensitization of C fiber, Aδ fiber, and Aβ fiber, respectively. The CPTs at 5, 250, and 2000 Hz were significantly increased on the vehicle-injected side of the diabetic mice compared with the normal mice, indicating hypoalgesia of the C fiber, Aδ fiber, and Aβ fiber in the diabetic mice. Four weeks after the transplantation of hDPSCs, these deficits in sensation were significantly improved on the hDPSC-transplanted side of the diabetic mice compared with the vehicle-injected side of the diabetic mice (*P* < 0.05). In contrast, the transplantation of hDPSCs in the normal mice did not alter the CPTs.

### Characterization of the hDPSCs at 4 weeks after transplantation

**Fig. 4:**
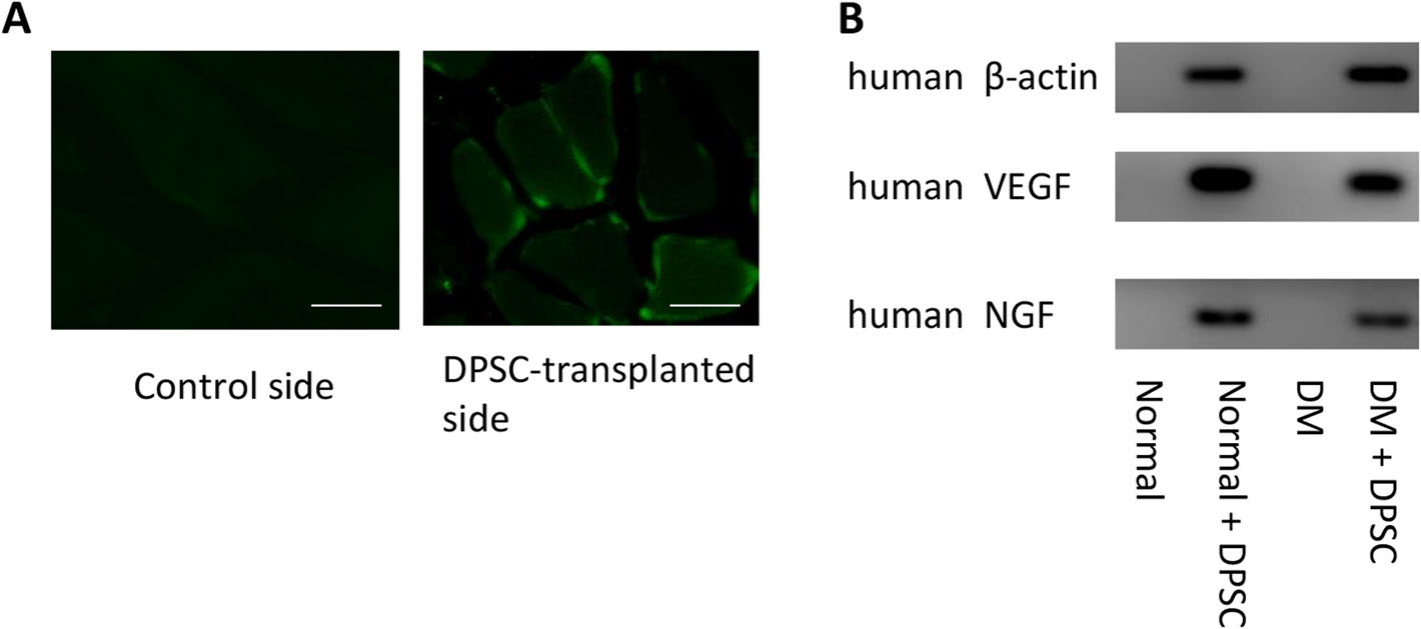
Localization of transplanted hDPSCs and expression of angiogenic and neurotrophic factors. **a** Four weeks after transplantation in the hindlimb skeletal muscles, the transplanted cells were stained with an antibody specific for human nuclei. Bar = 25 μm. **b** The mRNA expression of VEGF and NGF in the hindlimb skeletal muscles was assessed by real-time quantitative polymerase chain reaction with human probes. The products were observed via agarose gel electrophoresis with ethidium bromide staining

The transplanted hDPSCs were detected by immunohistological staining with an antibody specific for human nuclei. As shown in Fig. 4a, the hDPSCs transplanted into the skeletal muscles were localized around the muscle bundles at 4 weeks after transplantation. No human nuclei were observed on the contralateral side in either normal or diabetic mice.

### Transplanted hDPSC gene expression of angiogenic and neurotrophic factors

To confirm whether the transplanted hDPSCs localized to the hindlimb skeletal muscles and expressed angiogenic and neurotrophic factors, we investigated the expression of the human VEGF, NGF, and β-actin genes. As shown in Fig. 4b, the hDPSC-transplanted side of the gastrocnemius muscles expressed human VEGF, NGF, and β-actin mRNA, as visualized by agarose gel electrophoresis, in both the normal and diabetic mice. None of these human genes was expressed on the vehicle-injected side of the gastrocnemius muscles.

### Protein levels of human VEGF and human NGF in serum and hDPSC culture supernatants

To confirm the secretion of VEGF and NGF from hDPSCs, we evaluated the protein levels of VEGF and NGF in hDPSC culture supernatants using human VEGF and human NGF ELISA kits. The concentrations of VEGF and NGF in the hDPSC culture supernatant were 2.62 ± 0.42 ng/mL and 35.7 ± 4.23 pg/mL, respectively. In contrast, the serum concentrations of both VEGF and NGF were below the detectable range in both the normal and diabetic mice.

### hDPSC transplantation increases the capillary/muscle bundle ratio in diabetic mice

**Fig. 5:**
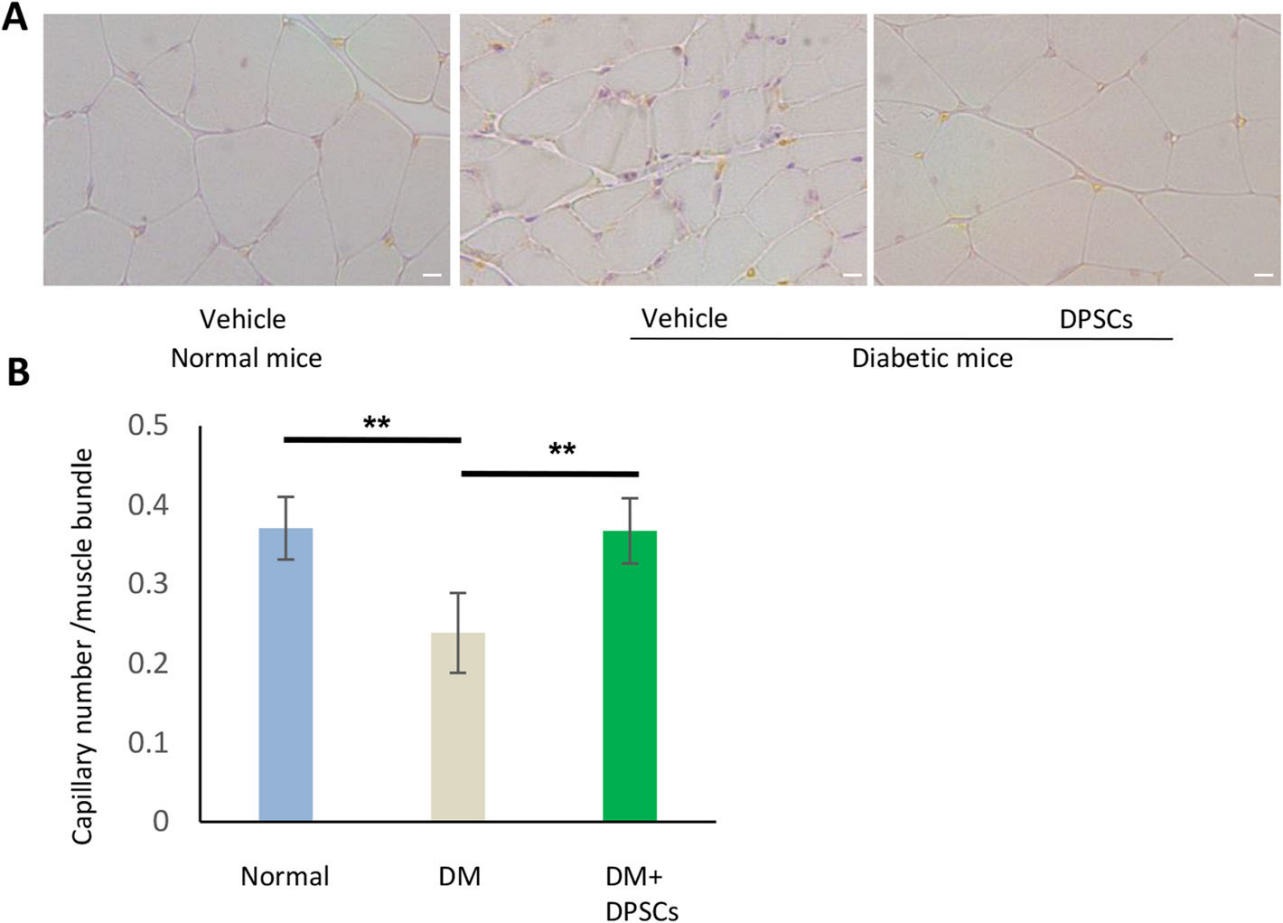
Capillary/muscle bundle ratio in hindlimb skeletal muscles. **a** Representative photomicrographs of histological gastrocnemius muscle sections from vehicle-injected normal mice, vehicle-injected diabetic mice, and hDPSC-injected diabetic mice. The capillaries were detected by immunostaining for PECAM-1. Bar = 50 μm. **b** Quantitative analyses of the capillary to muscle bundle ratio. The results are expressed as the mean ± SD (*n* = 4). **P < 0.01

Capillaries were visualized by PECAM-1 immunostaining (Fig. 5a). Quantitative analyses revealed that the capillary/muscle bundle ratio on the vehicle-injected side of diabetic mice was significantly reduced compared with that on the vehicle-injected side of normal mice (*P* < 0.01). Transplantation of hDPSCs significantly increased the number of capillaries in diabetic mice (*P* < 0.01) (Fig. 5b).

### The effect of hDPSC transplantation on MNCV and SNCV was negated by VEGF- and NGF-neutralizing antibodies

**Fig. 6:**
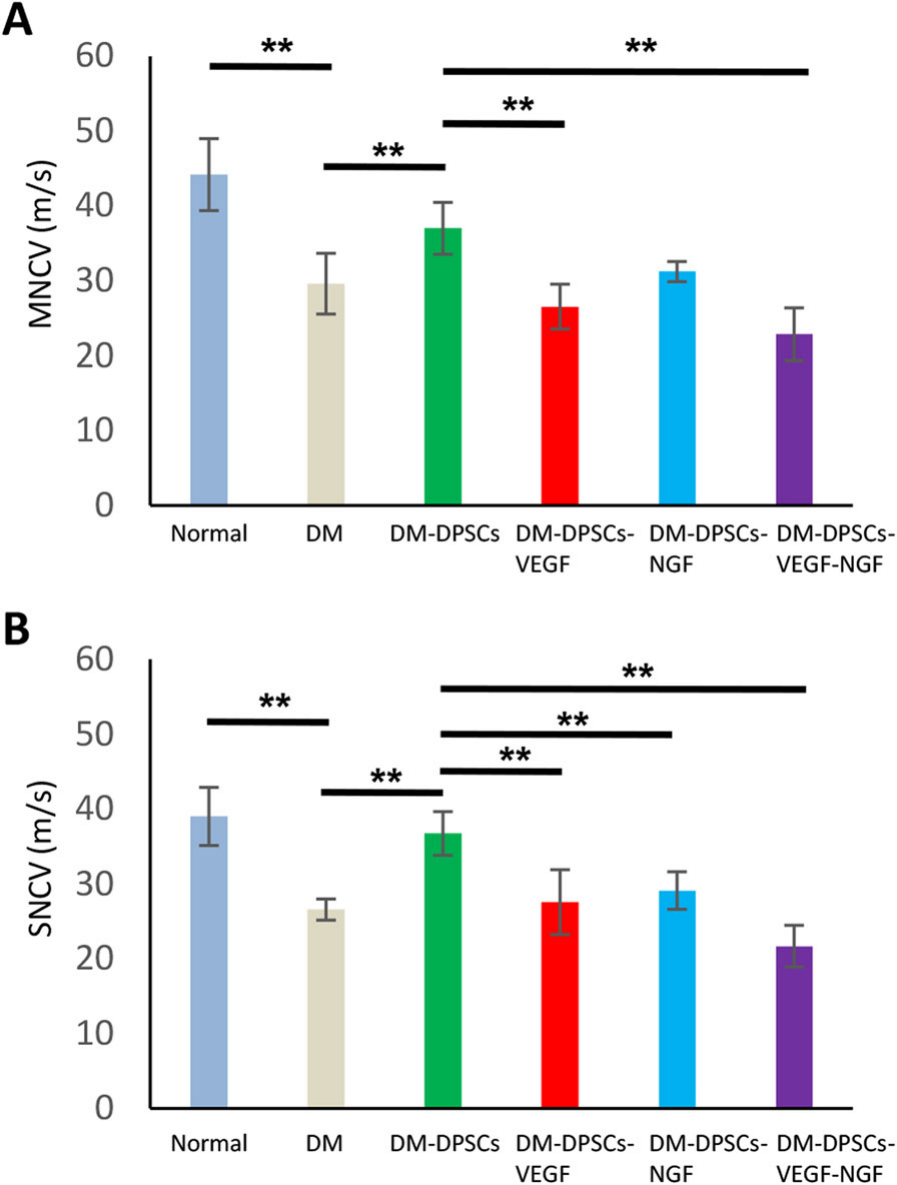
Inhibition of VEGF and NGF by specific neutralizing antibodies significantly suppressed the effects of hDPSC transplantation on the MNCV (**a**) and SNCV (**b**). The neutralizing antibodies against VEGF and NGF were continuously administered using an osmotic pump. The pump was inserted on the day of hDPSC transplantation. The results are expressed as the mean ± SD (*n* = 6). **P < 0.01

To clarify the effects of hDPSC-secreted angiogenic and neurotrophic factors on hDPSC transplantation, we continuously administered VEGF- and/or NGF-neutralizing antibodies to diabetic mice using an osmotic pump beginning on the day of hDPSC transplantation. MNCV and SNCV were measured 4 weeks after hDPSC transplantation. The blood glucose levels were significantly higher in all groups of diabetic mice compared with that of normal mice. VEGF- and/or NGF-neutralizing antibodies did not show significant changes in body weights nor blood glucose levels of diabetic mice (Supplemental figure S1). The delayed MNCV and SNCV on the vehicle-injected side of the diabetic mouse were significantly improved by hDPSC transplantation (*P* < 0.01), and the administration of VEGF- and NGF-neutralizing antibodies significantly suppressed the effects of hDPSC transplantation on the MNCV and SNCV (Fig. 6a, b).

## Discussion

**Fig. 7:**
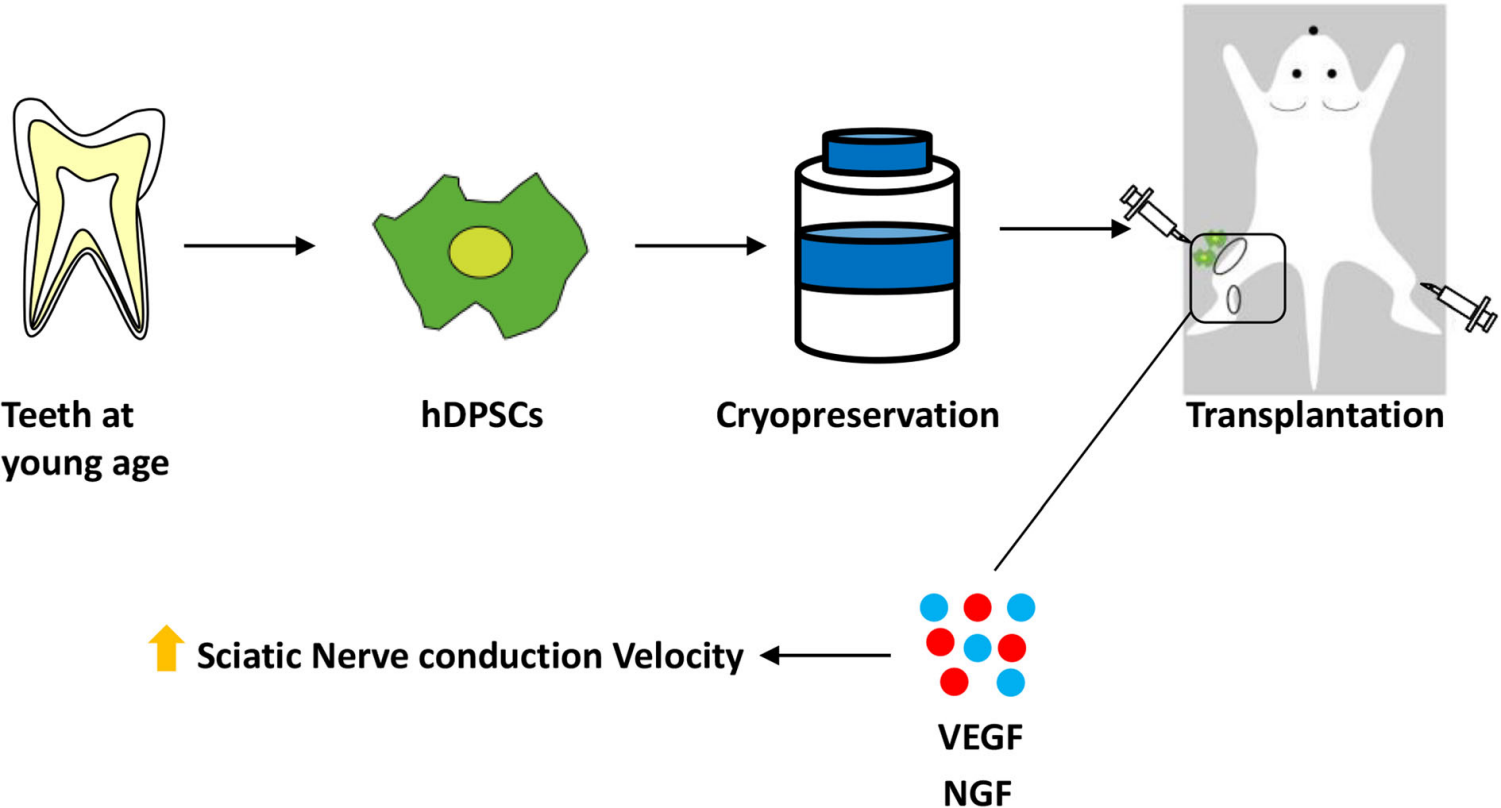
Schematic overview of the therapeutic effects of human DPSC transplantation on diabetic polyneuropathy

Here, we demonstrate the efficacy of using transplanted hDPSCs isolated from human extracted third molars to treat diabetic polyneuropathy in diabetic nude mice. In this study, we showed that transplanted hDPSCs ameliorated decreased nerve conduction velocity and nerve blood flow in diabetic mice. Transplantation of hDPSCs also improved the sensory disorder of small fibers (C fiber and Aδ fiber) and a large fiber (Aβ fiber). We further demonstrated that hDPSCs transplanted into the skeletal muscles were localized around the muscle bundles and produced human angiogenic and neurotrophic factors. The administration of neutralizing antibodies against VEGF and/or NGF negated the therapeutic effects of hDPSC transplantation on nerve conduction velocity, suggesting that these angiogenic and neurotrophic factors play a crucial role in hDPSC transplantation (Fig. 7).

DPSCs can be isolated from the dental pulp and show an MSC phenotype [20, 21]. In this study, hDPSCs were isolated from dental pulp tissues of the third molars and expressed CD29, CD73, CD90, and CD105 on their surface, which confirmed their derivation from mesenchymal stem cells. DPSCs are considered an attractive candidate for cell therapy because they can be isolated from the teeth extracted at a young age for orthodontic reasons and can be cryopreserved until use, as they maintain high bioavailability [2, 17]. hDPSCs were first derived from the human dental pulp in 2000 [22], and their therapeutic efficacy in cardiovascular diseases and spinal cord transection has been demonstrated [23–25]. A previous study demonstrated that DPSC transplantation into the skeletal muscles was superior to intravenous DPSC transplantation for the treatment of diabetic polyneuropathy [26]. Our results indicated that hDPSC transplantation in diabetic mice improved the sciatic nerve MNCV/SNCV and SNBF as well as sensory nerve thresholds detected by a CPT/LAB neurometer, which is now widely clinically used to measure the effects of analgesic drugs on and peripheral nerve functions in neuropathy [27]. After 12 weeks of diabetes, hypoalgesia at the Aβ fiber, Aδ fiber, and C fiber was observed in the diabetic mice, which showed recovery following hDPSC transplantation. In the hindlimb skeletal muscles, the capillary number was significantly decreased in diabetic mice compared with normal mice, which was improved by the transplantation of hDPSCs.

The advantages of cell therapy with hDPSCs are as follows: autologous transplantation; easy isolation from extracted teeth at a young age, without further invasion; and conservation of their original capability via cryopreservation. Because hDPSCs isolated at a young age, before the onset of diabetes, are not affected by hyperglycemia, they may be free of the stem cell dysfunction typical of diabetes.

We and others have revealed the therapeutic effects of progenitor/stem cell transplantation on diabetic polyneuropathy in rodent animal models [14–16]. However, the mechanisms by which progenitor/stem cell transplantation improve diabetic polyneuropathy are not clear. In this study, we demonstrated hDPSC localization around muscle bundles and human VEGF and NGF mRNA expression in transplanted skeletal muscles, indicating that a subset of hDPSCs remained at the transplanted site and expressed these angiogenic and neurotrophic factors at 4 weeks after transplantation. The hDPSC culture experimental results confirmed that the human VEGF and human NGF proteins were secreted from hDPSCs and were consistent with those of other studies. Mead et al. reported that hDPSCs secreted higher levels of both neurotrophic factors, including NGF and brain-derived neurotrophic factor, and VEGF than human bone marrow- and human adipose tissue-derived MSCs [28]. Another study demonstrated that DPSCs induced angiogenesis via the VEGFR2 receptor [29]. We confirmed that the concentration of the human VEGF protein in hDPSC culture media was 74-fold higher than that of the human NGF protein. Our previous study demonstrated that the VEGF gene expression in cultured rat DPSCs was more than 20-fold higher than that of NGF [30].

We considered that the VEGF and NGF secreted from hDPSCs had mainly local effects and that their systemic effects were relatively weak because the serum concentrations of VEGF and NGF as determined by ELISA were below the detectable range in both normal and diabetic mice. The studies of nerve conduction velocities and nerve blood flow support the local effects of hDPSCs, as the MNCV, SNCV, and SNBF on the hDPSC-transplanted side were significantly improved compared with those on the vehicle-injected side in diabetic mice. The capillary number in the gastrocnemius was also significantly increased on the hDPSC-injected side compared with the vehicle-injected side in the diabetic mice. Our previous study confirmed the local effects of rat DPSCs by demonstrating that the impaired MNCV, SNCV, and SNBF were nearly the same between non-treated diabetic rats and diabetic rats transplanted with DPSCs on the vehicle-injected side [30].

VEGF is recognized as not only an angiogenic factor but also a neuroprotective factor [31]. A clinical trial involving the VEGF gene demonstrated the therapeutic effects of VEGF on diabetic polyneuropathy [32]. NGF binds to p75 and trk A and affects small sensory and autonomic nerve fibers. Reduced NGF expression was reported in diabetic animals, and the administration of NGF ameliorated pain sensation in diabetic patients [33, 34]. Although there may be many cytokines which affect the therapeutic effects of hDPSC transplantation on diabetic polyneuropathy, we confirmed that the effect of hDPSC transplantation on MNCV and SNCV in diabetic mice was negated by human VEGF- and human NGF-neutralizing antibodies. Since the VEGF-neutralizing antibody had less than 20% cross-reactivity with mouse VEGF, and the NGF-neutralizing antibody had more than 50% cross-reactivity with mouse NGF, we could not completely nullify the effects of these neutralizing antibodies on mouse endogenous VEGF and NGF. However, these results suggest that angiogenic and neurotrophic factors play an important role in the effect of hDPSC transplantation. Further study is required to address this issue.

## Conclusions

In summary, these findings suggest that hDPSCs may have a role in the future in treating diabetic polyneuropathy. hDPSC transplantation ameliorated not only nerve conduction velocity, nerve blood flow, and sensory disorder but also the capillary/muscle bundle ratio in the hindlimb skeletal muscles. The results of this study performed using neutralizing antibodies suggest the crucial roles of VEGF and NGF in hDPSC transplantation.

## Supplementary information

**Additional file 1: Figure S1.** Body weights **(a)** and blood glucose concentration **(b)** of normal and diabetic nude mice some of which were treated with a VEGF neutralizing antibody and /or a NGF neutralizing antibody soon after hDPSC transplantation.

## Data Availability

All data generated and/or analyzed during this study are included in this published article. Data sharing is not applicable to this article, as no datasets were generated or analyzed during the current study. However, the data that support the findings of this study are available from the corresponding author upon reasonable request.

## Abbreviations

CPT: Current perception threshold
DPN: Diabetic polyneuropathy
DPSC: Dental pulp stem cell
EPCs: Endothelial progenitor cells
FACS: Fluorescence-activated cell sorting
hDPSC: Human dental pulp stem cell
hDPSC-CM: hDPSC-conditioned medium
MNCV: Motor nerve conduction velocity
NGF: Nerve growth factor
PCR: Polymerase chain reaction
PECAM-1: Platelet endothelial cell adhesion molecule-1
qRT-PCR: Real-time quantitative polymerase chain reaction
SNBF: Sciatic nerve blood flow
SNCV: Sensory nerve conduction velocity
STZ: Streptozotocin
VEGF: Vascular endothelial growth factor
α-MEM: Alpha modification of Eagle’s medium
