# Supplementary figures and images for: Transplantation of human dental pulp stem cells ameliorates diabetic polyneuropathy in streptozotocin-induced diabetic nude mice: the role of angiogenic and neurotrophic factors
Source: Stem Cell Res Ther. 2020 Jun 16;11:236. doi: 10.1186/s13287-020-01758-9 (PMC7298811; doi:10.1186/s13287-020-01758-9)

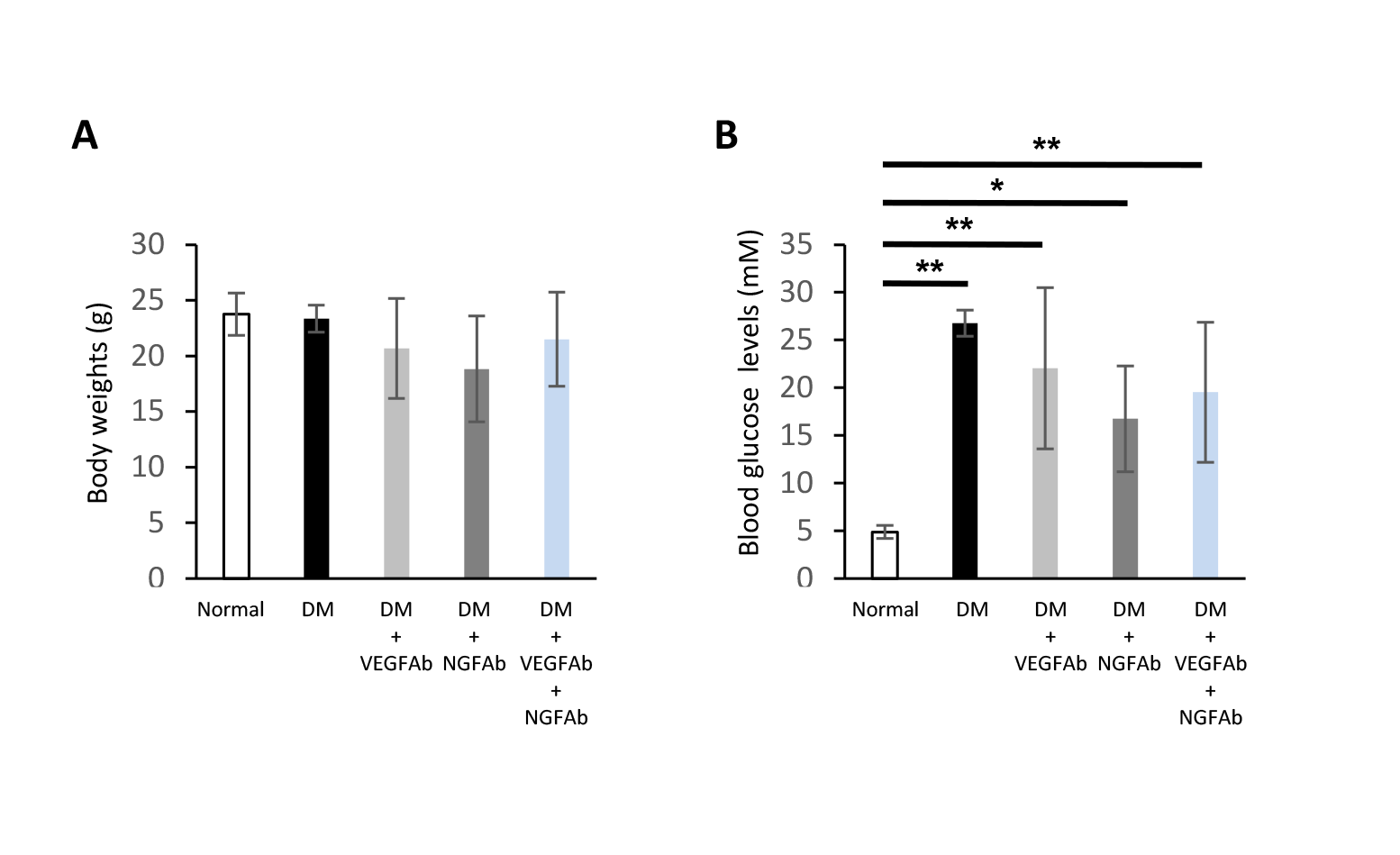

Supplement: Supplementary file 1 — Additional file 1: Figure S1. Body weights (a) and blood glucose concentration (b) of normal and diabetic nude mice some of which were treated with a VEGF neutralizing antibody and /or a NGF neutralizing antibody soon after hDPSC transplantation. [file 13287_2020_1758_MOESM1_ESM.tif]
